# Physical controllability of complex networks

**DOI:** 10.1038/srep40198

**Published:** 2017-01-11

**Authors:** Le-Zhi Wang, Yu-Zhong Chen, Wen-Xu Wang, Ying-Cheng Lai

**Affiliations:** 1 School of Electrical, Computer, and Energy Engineering, Arizona State University, Tempe, AZ 85287, USA; 2 Department of Systems Science, School of Management and Center for Complexity Research, Beijing Normal University, Beijing 100875, China; 3 Department of Physics, Arizona State University, Tempe, AZ 85287, USA

## Abstract

A challenging problem in network science is to control complex networks. In existing frameworks of structural or exact controllability, the ability to steer a complex network toward any desired state is measured by the minimum number of required driver nodes. However, if we implement actual control by imposing input signals on the minimum set of driver nodes, an unexpected phenomenon arises: due to computational or experimental error there is a great probability that convergence to the final state cannot be achieved. In fact, the associated control cost can become unbearably large, effectively preventing actual control from being realized physically. The difficulty is particularly severe when the network is deemed controllable with a small number of drivers. Here we develop a physical controllability framework based on the probability of achieving actual control. Using a recently identified fundamental chain structure underlying the control energy, we offer strategies to turn physically uncontrollable networks into physically controllable ones by imposing slightly augmented set of input signals on properly chosen nodes. Our findings indicate that, although full control can be theoretically guaranteed by the prevailing structural controllability theory, it is necessary to balance the number of driver nodes and control cost to achieve physical control.

The past few years have witnessed great progress toward understanding the linear controllability of complex networks^1^,^2^,^3^,^4^,^5^,^6^,^7^,^8^,^9^,^10^,^11^,^12^,^13^,^14^,^15^,^16^,^17^,^18^,^19^,^20^,^21^,^22^,^23^,^24^,^25^,^26^,^27^,^28^. Given a linear and time-invariant dynamical system, the traditional approach to assessing its controllability is through the Kalman rank condition^29^. However, for a complex network, it is difficult to test, both mathematically and computationally, the Kalman rank condition directly to determine the optimal configuration for control input signals^1^ due to the typically large network size and the complex spectrum of network topology. To overcome this difficulty, Liu *et al*. proposed in their pioneering work^4^ to exploit Lin’s classic theory of structural controllability^30^. In this framework, the fundamental issue is to determine the minimum number of controllers required to steer the whole networked system from an arbitrarily initial state to an arbitrarily final state in finite time. It was proved and demonstrated^4^ that, for directed complex networks, their structural controllability can be established via the maximum matching algorithm^31^,^32^,^33^. In particular, based on Lin’s theory, one can determine the maximally matched set of nodes, where each and every unmatched node requires an external control signal. An equivalent optimization procedure was developed for undirected networks to determine the minimum dominating set of nodes^6^. The structural controllability framework also served the base to address an array of issues such as edge dynamics^8^, lower and upper bounds of energy required for control^7^, control centrality^34^, optimization^5^, effects of the density of in/out degree nodes^14^, and scaling of energy cost^27^. In addition, based on the classic Popov-Belevitch-Hautus (PBH) rank condition^35^ from traditional control engineering, a variant of the structural-controllability theory, an exact controllability framework was developed^10^ which is universally applicable to all kinds of complex networks: directed or undirected, weighted or unweighted. In terms of applications, the structural controllability framework has been used to characterize protein interaction networks to determine the key proteins responsible for certain biological functions^16^.

In both structural and exact controllability frameworks, the focus is to determine the minimum number of control signals, denoted by *N*_*D*_, for complex networks of various topologies. However, we have encountered an unexpected difficulty when using the minimal set given by either structural or exact controllability theory to carry out *actual* control of the network: convergence to the final state. In particular, given a network, once *N*_*D*_ is determined, we can determine the specific control signals to be applied at various unmatched nodes by using the standard linear systems theory^36^. The surprising phenomenon is that, quite often, actual control of the system cannot be achieved computationally in the sense that, in any finite time, the system cannot be driven from an arbitrarily initial state to an arbitrarily final state. We believe that this difficulty is fundamental, as we were not able to remove or even mitigate the problem of divergence despite extensive and systematic computational efforts in implementing various ways to optimize the numerical algorithm. This difficulty in realizing actual control persists for a large number of model and real world networks. While somewhat unsettling, the issue prompts us to hypothesize that the existing controllability frameworks are merely *mathematical*, as the implementation of actual control would often require infinite precision computations and, more seriously, an infinite amount of energy. To make the notion of controllability of complex networks meaningful, the issue of *physical* controllability must be addressed.

In this paper, we develop a physical controllability framework for complex networks to address whether actual control can be achieved in an experimentally or computationally feasible way. Given a complex network, we first use the structural controllability theory^4^ to determine *N*_*D*_ and a set of unmatched nodes to which control signals are to be applied. Then, with a given pair of arbitrarily initial and final states as well as a finite control time, we calculate the optimal control signals^36^ and evolve the whole networked system, which is essentially a linear dynamical system under external driving, to determine whether the system can be driven from the initial state to the final state in the given amount of time. During this process, the energy required for control can be calculated through the standard formula in linear systems theory^36^,^37^, which expresses the energy as the integral of the product of a number of matrices, including the inverse of the positive-definite, symmetric Gramian matrix. Freedom in choosing the initial and final states and independent network realizations render feasible a statistical analysis of the control process. We find that, typically, there are two cases, depending on whether the network can be physically controlled. For the physically controllable case, the whole system, starting from the chosen initial condition, can actually converge to the final state in the prespecified time *within a predefined precision*. In this case, the Gramian matrix is well-behaved, meaning that both its condition number and the energy are not unrealistically large. For the physically uncontrollable case, the system cannot reach the final state within the predefined precision in the given time. In such a case, the Gramian matrix is singular in the sense that its condition number can be arbitrarily large, so is the corresponding energy. Increasing the precision of the computation, e.g., by using special simulation packages with round-off error orders of magnitude smaller than that associated with the conventional double-precision computation, would convert a few uncontrollable cases into controllable ones, but vast majority of the uncontrollable cases remain unchanged.

The main result of this paper is a proposal of a general, probabilistic measure to characterize the physical controllability for complex networks of arbitrary topology. For physically uncontrollable networks, it is important to develop effective strategies to make them physically controllable. To accomplish this goal, we gain insights by calculating the control energy for a bidirectional 1D chain and obtaining an analytical relation between energy *E* and chain length *L*. We then apply the result to general networked systems based on the idea of longest control chain (LCC)^27^. Optimization strategies can be derived to decrease the control energy drastically. In fact, if the system is physically uncontrollable, a viable way to make it controllable is to increase the number of control signals beyond *N*_*D*_. Our framework of physical controllability thus contains the following essential ingredients: (1) *N*_*D*_, the minimum number of control signals determined by the existing mathematical controllability frameworks, (2) a measure of physical controllability, (3) control energy *E* determined by the Gramian matrix, and (4) augmentation of *N*_*D*_ for physically uncontrollable networks. The existing mathematical controllability theories^4^,^10^ thus provide a base for our physical controllability framework. The quantity *N*_*D*_, on which the mathematical controllability theories focus, can effectively be regarded as the *lower bound* of the actual number of control signals required. To realize physical control, depending on the specific system and control settings, either *N*_*D*_ control signals suffice or substantially more signals are needed.

## Results

### Definition of physical controllability

We consider the standard setting of a linear dynamical system subject to control input^1^,^4^,^10^:


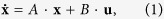


where **x** = [*x*_1_(*t*), …, *x*_*N*_(*t*)]^*T*^ is a vector of dynamical variables of the entire network, **u** = [*u*_1_(*t*), …, *u*_*M*_(*t*)]^*T*^ is a vector defining the set of control input signals, *A* = {*a*_*ij*_}_*N*×*N*_ is the adjacency matrix with *N* being the number of nodes in the network, and 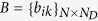 is control input matrix specifying the set of *N*_*D*_ “driver” nodes^4^, each receiving a control signal that corresponds to one component of the control vector **u**. From the linear systems theory, optimal control of a linear dynamical network in the sense of minimized energy cost can be achieved when the input control signals **u**_*t*_ are chosen as^36^,^37^: 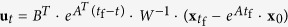, where


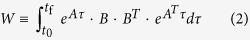


is the Gramian matrix, a positive-definite and symmetric matrix^36^, which serves as the base to determine quantitatively if a system is actually controllable. In particular, the system is controllable only when *W* is nonsingular (invertible) for given control precision^36^,^37^. With the control input signal **u**, the energy cost is^36^


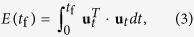


where control is initiated at *t* = 0 and ended at *t* = *t*_f_.

To present concrete evidence for the existence of physically uncontrollable networks, we use the Erdos-Renyi (ER) type of directed random networks^38^ and the Barabási-Albert (BA) type of directed scale-free networks^39^ with a single parameter *P*_b_. The meaning of *P*_b_ is the following. Given a pair of linked nodes, *i* and *j*, the probability that the link points from the smaller-degree node to the larger-degree one is *P*_b_, and the probability in the opposite direction is 1 − *P*_b_. The link direction is chosen randomly if *i* and *j* have the same degree. To determine the set of driver nodes, we use the maximum-matching algorithm^30^, which gives the control matrix *B*. For each combination of *A* and *B*, we first randomly choose the initial and final states. We then calculate the corresponding Gramian matrix *W*, its condition number, the input signal **u**_*t*_, the actual final states 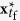, and finally the control energy *E(t*_f_). Repeating this process for each and every independent network realization in the ensemble enables an extensive statistical analysis of the control process.

Mathematically, if the Gramian matrix *W* is singular, the energy diverges. Through extensive and systematic numerical computations, we find that, even when *W* is non-singular in the mathematical sense, for typical complex networks its condition number can be enormously large, making it effectively singular as any physical measurement or actual computation must be associated with a finite precision. Say in an experiment the precision of measurement is *ε*. In a computational implementation of control, *ε* is effectively the computer round-off error. Consider the solution vector **X** of the linear equation: *W* · **X** = **Y**, where **Y** is a known vector. Let *C*_*W*_ be the condition number of *W*. The accuracy of the numerical solution of **X**, denoted by *e*_*X*_ = 10^−*k*^ (*k* is a positive integer), is bounded by the product between *C*_*W*_ and *ε*^40^. We see that, if *C*_*W*_ is larger than 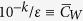, it is not possible to bring the system to within 10^−*k*^ of the final state at finite control cost, so physically control cannot be achieved in finite time.

**Figure 1:**
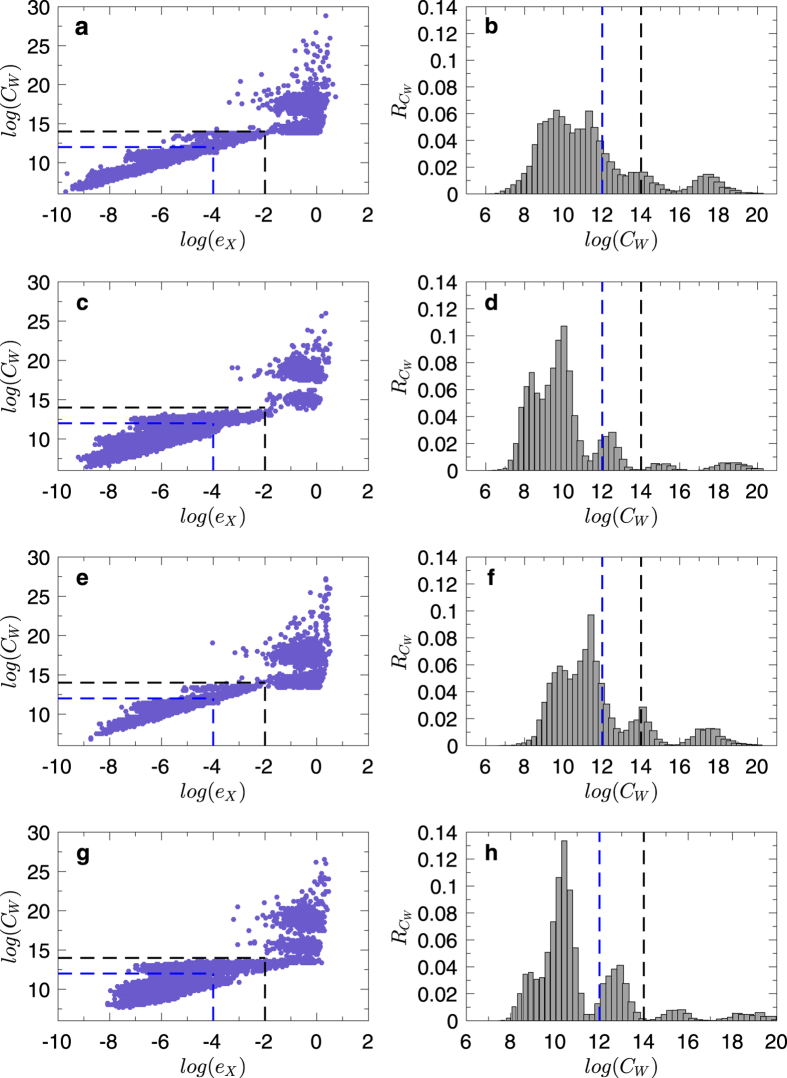
Condition number *C*_*W*_ versus control precision *e*_*X*_ for random and scale-free networks. Network size is *N* = 100 for (**a**–**d**) and 200 for (**e**–**h**), average degree is 〈*k*〉 = 6 for ER random networks [(**a**), (**b**), (**e**) and (**f**)] and 8 for BA scale-free networks [(**c**), (**d**), (**g**) and (**h**)]. Directional link probability between any pair of nodes is *P*_b_ = 0.1. Panels (**a**), (**c**), (**e**) and (**g**) show the scaling relation between the condition number *C*_*W*_ and the control precision *e*_*X*_. Panels (**b**), (**d**), (**f**) and (**h**) show the fraction *R*_*CW*_ of the networks with a certain *C*_*W*_ number. The scaling relation holds within some *C*_*W*_-*e*_*X*_ region with boundaries specified as the black dashed lines. The *e*_*X*_ values are not physically meaningful outside the boundaries that are defined according to the precision limit of computation. The thresholds of *C*_*W*_ and *e*_*X*_ used in the computations are 10^12^ and 10^−4^, respectively, which are indicated as the blue dashed lines. The threshold values are chosen to lie within the physical boundaries so that the calculations for all *C*_*W*_ values are meaningful.

For a large number of networks drawn from an ensemble of networks with a pre-defined topology, the condition numbers of their Gramian matrices are often orders of magnitude larger than 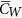. Figure 1 shows the correlation between the condition number *C*_*W*_ and the control error *e*_*X*_. We observe that, within a certain range of *C*_*W*_, an approximate scaling relation exists between *C*_*W*_ and *e*_*X*_, as shown in panels (a, c, e, g). However, the scaling disappears outside the range where the Gramian matrix *W* is ill conditioned, leading to considerable errors when computing the matrix inverse. In principle, the scaling regime can be extended with improved computational precision, but not indefinitely. For the networks with an ill conditioned Gramian matrix, not only is the control vector unable to drive the system to the target state, but the associated energy can be extremely large. These observations suggest the following criterion to define physical controllability in terms of the control energy cost: a network is controllable with respect to a specific control setting if and only if the condition number of its Gramian matrix is less than 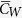, a critical number determined by both the measurement or computational error and the required precision of control. For a given set of network parameters (hence a given network ensemble) and control setting, the probability that the condition number of the Gramian matrix is less than 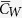, 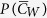, can effectively serve as a quantitative measure of *physical controllability*. Increasing the precision of the computation, e.g., by using special simulation packages with round-off error orders of magnitude smaller than that associated with the conventional double-precision computation, would convert a few uncontrollable cases into controllable ones, but vast majority of the uncontrollable cases remain unchanged.

Note that, physical controllability is characterized by the condition number of the Gramian matrix *W*, which is defined by the adjacency matrix *A*, the control matrix *B*, and the control time from *t*_0_ to *t*_f_. The adjacency matrix *A* totally defines the structure of the underlying network and, in the absence of control, solely determines the evolution of the system from an initial state. The purpose of control is to design the control matrix *B* so that the Gramian matrix is numerically to ensure that the system is physically controllable, which can be accomplished regardless of whether the matrix *A* is stable or unstable.

We also note that, in a linear dynamical system, the Gramian matrix *W* is determined by the network structure, the control configuration, and the control time; it does not depend on the dynamical trajectory. As a result, additive noise of reasonable amplitude does not affect the physical controllability of the network.

### Structural controllability does not imply physical controllability

**Figure 2:**
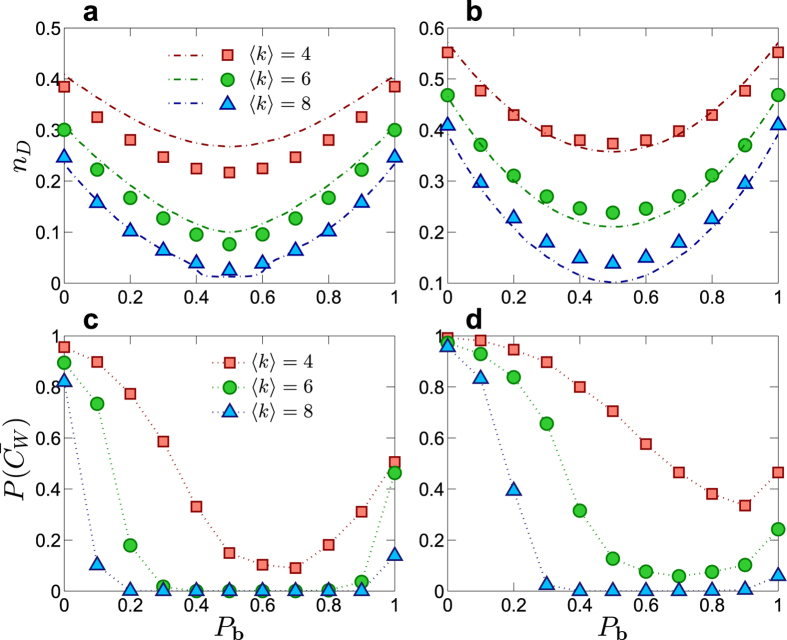
Structural and physical controllability measures in directed networks. Structural controllability measure *n*_D_ versus directional edge probability *P*_b_ for (**a**) ER random networks and (**b**) BA scale-free networks of size *N* = 1000 and three values of the average degree (〈*k*〉 = 4, 6, and 8). The dash-dotted lines represent the results obtained from the cavity method^4^,^5^, and the squares, triangles, and circles are simulation results from the maximum matching algorithm^4^. (**c**,**d**) Measure of physical controllability 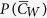 for ER random and BA scale-free networks of size *N* = 100, respectively, where 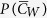 is the probability that the condition number of the Gramian matrix is less than some physically reasonable threshold value. Comparing (**a**) with (**c**), or (**b**) with (**d**), we observe the striking phenomenon that, in the parameter regime where the number of driver nodes is minimized so that the corresponding networks are deemed to be most structurally controllable, they are physically uncontrollable. The phenomenon persists regardless of the network size and type. All nodes are self-loop free. The qualitative behavior is robust against variations in the value of 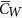.

We present evidence that structural and physical controllabilities are not necessarily compatible with each other. Figure 2(a,b) show the percentage of driver nodes, *n*_D_ ≡ *N*_D_/*N*, versus the directional link probability *P*_b_. We see that *n*_D_ is minimized for *P*_b_ = 0.5, indicating a maximal (optimal) level of structural controllability because only a few control signals are needed to control the whole network^4^. But can physical controllability be achieved in the same parameter regime where structural controllability is optimized? Figure 2(c,d) show the corresponding physical controllability 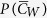 versus the network parameter *P*_b_. We see that, in both regimes of small and large *P*_b_ values where structural controllability is weak [corresponding to relatively high values of *n*_D_ in Fig. 2(a,b)], the physical controllability is relatively strong. In the regime of small *P*_b_ values, most directed links in the network point from small to large degree nodes. In this case, the network is more physically controllable, in agreement with intuition. The striking result is that, in the regime of intermediate *P*_b_ values (e.g., *P*_b_ around 0.5) where the number of driver nodes to control the whole network is minimized so that structural controllability is regarded the strongest, the physical controllability is in fact the weakest, as the probability of the condition number 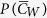 being small is close to zero. For example, for the random networks in Fig. 2(c), for 〈*k*〉 = 4, the minimum value of 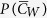 is only about 0.1 for *P*_b_ ≈ 0.6, while for 〈*k*〉 = 6 and 〈*k*〉 = 8, the minimum values are essentially zero. Surprisingly, near zero values of 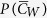 occur in a wide range of the parameter *P*_b_, e.g., [0.3, 0.8] and [0.2, 0.9] for 〈*k*〉 = 6 and 〈*k*〉 = 8, respectively, as shown in Fig. 2(c). This indicates that the network is physically uncontrollable for most cases where structural controllability is deemed to be strong. BA scale-free networks behave similarly, as illustrated in Fig. 2(d). Another finding from Fig. 2 is that *N*_D_ is symmetric about *P*_b_ = 0.5, but the symmetry is broken for 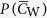, indicating that there is no simple linear correlation between *N*_D_ and 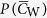. It is thus necessary to find the fundamental structural properties responsible for the smallness of 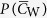. Through a detailed analysis of the energy cost associated with controlling a simple one-dimensional chain and a double chain network (**Methods**) and of the energy scaling^27^, we identify the longest control chains (LCCs), the shortest paths through which the control energy is “flowed” to all nodes in the network, as the fundamental structural component responsible for the control energy. The longer the LCCs, the more singular the Gramian matrix, and the smaller the probability 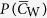. The maximal LCC is effectively the *control diameter* of the network^27^.

### Physical controllability of an electrical circuit network and a strategy to balance control energy and extra inputs

**Figure 3:**
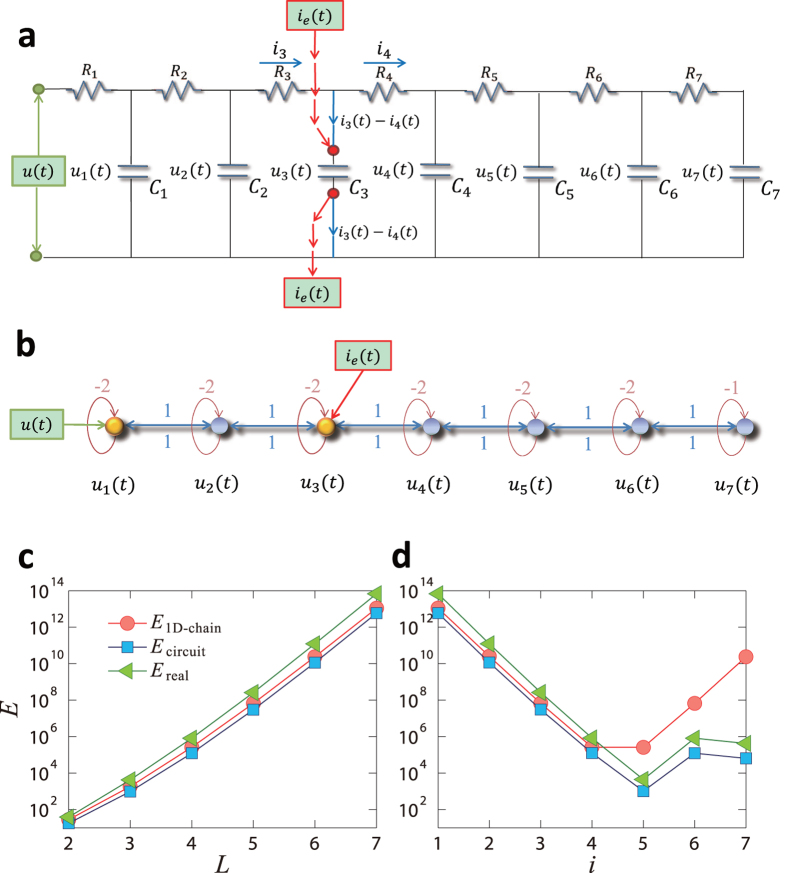
Illustration of parallel R-C circuit and optimization of control energy. (**a**) A cascade parallel R-C circuit with *L* = 7 resistors (*R*_1_, *R*_2_, …, and *R*_*L*_, each of resistance 1Ω) and 7 capacitors (*C*_1_, *C*_2_, …, and *C*_*L*_, each of capacitance 1*F*). External voltage input *u(t*) is applied from the left side of the circuit, and the voltage of capacitor *C*_*i*_ is *u*_*i*_(*t*)(1 ≤ *i* ≤ *L*). An extra external current input *i*_e_(*t*) serves as a redundant control input injected into the capacitor *C*_3_, where *i*_3_ and *i*_4_ denote the currents through resistors *R*_3_ and *R*_4_, respectively. In absence of the extra current input, *i*_3_(*t*) − *i*_4_(*t*) is the current through the branch of *C*_3_. (**b**) Network representation of the circuit in (**a**) as a bidirectional 1D chain network of seven nodes, where the external voltage input *u(t*) is injected into node 1 (yellow driver node, the controller). The dynamical state of node *i* is described by the voltage of its capacitor, *u*_*i*_(*t*). Links (blue) between nodes are bidirectional and have uniform weight 1 in either direction. Each node has a self-link (red) of weight −2, except the ending node (node 7) whose self-link has weight −1. The extra external current input *i*_e_(*t*) serves as a redundant control input injected into node 3 of the network in (**b**). Now there are two driver nodes (yellow) in the network, nodes 1 and 3. (**c**) Energy required for controlling a unidirectional chain (red circle) and the corresponding circuit (blue square) as well as the dissipated energy (green triangle) of the circuit calculated from Eq. (4) versus chain length *L*. (**d**) Control and dissipated energies in presence of a redundant control signal to node *i (i* > 1), which breaks the chain into two subchains of lengths *i* and *L* − *i*, respectively.

To further illustrate the concept of physical controllability, we consider a real one-dimensional cascade parallel RC circuit network, as schematically illustrated in Fig. 3(a). The network can be represented by a bidirectional 1D chain with self-loops for all the nodes, as shown in Fig. 3(b) (see **Methods**). The network size can be enlarged, say by one unit, by attaching an additional branch of resistor and capacitor at the right end of the circuit. The state *u*_*i*_(*t*) of node *i* at time *t* is the voltage of capacitor *i*, and the input voltage *u(t*) represents the control signal. The purpose of control is to drive the voltages of the capacitors from a set of values to another within time *t*_f_ through the input voltage *u(t*). The control energy can then be calculated by Eq. (3). The actual energy dissipated in the circuit during the control process is given by


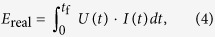


where *U(t*) ≡ *u(t*) and *I(t*) are the input voltage and current at time *t*, respectively, and *E*_real_ is in units of Joule. By making the circuit equivalent to a 1D chain network, we have three types of energy: the control energy of the actual circuit calculated from Eq. (3), the dissipated energy of the circuit from Eq. (4), and the control energy of the 1D equivalent network. Figure 3(c) shows that the control energy and the dissipated energy of the circuit do not differ substantially from the energy calculated from the unidirectional 1D chain. Among the three types of energy, the energy cost associated with the control process calculated from Eq. (4) is maximal.

Our extensive computations reveal that many structurally controllable networks are not physically controllable due to a combination of the ill-conditioned Gramian matrix and the finite computational or experimental error. Our analysis of the chain model (**Methods**) suggests a simple but effective strategy to reduce the energy significantly so as to enhance the physical controllability of the network: to place extra control signals along the LCCs to break the chains into shorter subchains. (In **Methods**, we show how the redundant control input can be planted in a circuit network.) To be illustrative, we consider a unidirectional 1D chain and add an extra control input at the *i*th node. As shown in Fig. 3(d), the magnitude of the control energy is reduced dramatically. The optimal location to place the extra control should be near in the middle of the chain so as to minimize the length of the LCC using a minimal number of extra control signals. In Fig. 3(d), the red circles represent a 1D chain and indicate that this simple strategy of adding one redundant control signal near the middle can reduce dramatically the required energy. For the circuit network in Fig. 3, the redundant control input can be realized by inducing external current input into a capacitor. In Fig. 3(d), the real energy is represented by green triangles, which reaches the minimum when the extra input is putting around the middle. Applying a single redundant control input can thus be an extremely efficient strategy to make the one-dimensional chain network physically controllable.

### Control energy optimization of complex networks

**Figure 4:**
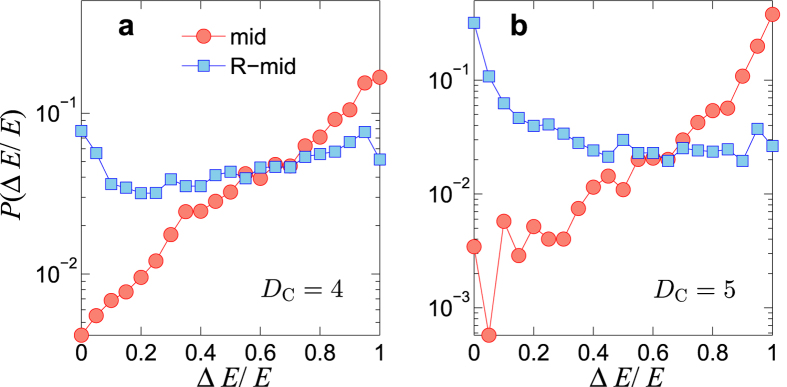
Effects of redundant control inputs. (**a**) For control diameter *D*_C_ = 4, distribution of the normalized energy reduction Δ*E*/*E* with redundant control in an ensemble of 10000 ER-random networks (〈*k*〉 = 6, *P*_b_ = 0.1). Results from the LCC-breaking optimization and random control augmentation are marked by “mid” (red circles) and “R-mid”(blue squares), respectively. For each network, a corresponding number of additional random control inputs are applied to the system 10 times to average out the statistical fluctuations. Panel (**b**) shows the Δ*E*/*E* distributions for networks with control diameter *D*_C_ = 5.

For a complex network, there often exist multiple LCCs, requiring multiple redundant control inputs. With insights from the RC circuit example, we see that a strategy is to place one redundant control input at the middle of each LCC. In this case, each LCC in the network is broken into two subchains. Figure 4(a) shows the effect of this optimization strategy on the energy distribution. For comparison, the same number of redundant control inputs are also applied randomly throughout the network. The reduction ratio between the control energy under optimization strategy, Δ*E*, and the original control energy *E* characterizes the effectiveness of the optimization process. In particular, if the distribution of Δ*E*/*E* is concentrated on large values of Δ*E*/*E*, then the corresponding optimization strategy can be deemed to be effective. As shown in Fig. 4(a), for relatively large Δ*E*/*E* values, *P*(Δ*E*/*E*) as a result of optimization has values that are systematically larger than those under random control signal augmentation, while the opposite situation is observed for regions with relatively smaller Δ*E*/*E*. Thus, our optimization strategy outperforms the random strategy. The networks requiring proper optimization to be physically controlled are typically those with large control diameters. Figure 4(b) show that this is indeed the case: for networks with larger values of *D*_C_, the performance of our optimization strategy is significantly better than that with random placement of extra controllers.

## Discussion

As stated in ref. 4, the ultimate proof that one understands a complex network completely lies in one’s ability to control it. However, we find that strong structural controllability is no guarantee that the network can be physically controlled. To resolve this paradox, We develop a physical controllability framework in terms of the control energy cost and the number of external input signals. To illustrate the framework, we focus on the situation where the structural controllability theory yields a minimum number of external input signals required for full control of the network, and determine whether in these situations the control energy is affordable so as to realize actual control. Our systematic computations and analysis reveal a rather unexpected phenomenon: due to the singular nature of the control Gramian matrix, in the parameter regimes where optimal structural controllability is achieved in the sense that the number of driver nodes is minimized, energy cost can be physically impossible to accommodate. To obtain a systematic understanding, we focus on a bidirectional 1D chain and study the relationship between energy and chain length. We then apply the 1D chain model to complex networks based on the idea of LCCs. In fact, the simple chain model captures the scaling behavior of energy distribution found in random networks^23^,^27^. The chain model also provides a guiding principle to articulate optimization strategies to reduce the control energy, which are tested using a RC circuit network and model complex networks.

An intuitive picture of the interrelation between mathematical controllability^4^,^10^ and our physical controllability is the following. In a fictitious world where the Gramian matrix is not singular (regardless of its condition number) and the computer round off or experimental errors are absolutely zero, using *N*_*D*_ controllers as determined by the structural controllability theory can bring the networked system from any initial state to any final state in a given time. However, in the physical world, the inevitable measurement or computational errors will have a devastating consequence in the execution of actual control as the Gramian matrix is typically effectively singular with an arbitrarily large condition number. The dynamical interplay between the error and the singular Gramian matrix makes the system uncontrollable in the sense that it cannot be driven to the final state in finite time within the desirable precision and the energy required in the process diverges. Often, to realize physical control, many more control signals than those determined by the structural controllability theory are needed.

Our work indicates the difficulty of achieving actual control of complex networks associated with even linear dynamics. Although the mathematical controllability theories^4^,^10^ offer theoretically justified frameworks to guide us to apply external inputs on a minimum set of driver nodes, when we implement control to steer a system to a desired state, the energy consumption is likely to be too large to be affordable. For nonlinear dynamical networks, we continue to lack a general controllability framework and an understanding of required control energy, although progress has been made^22^,^41^,^42^,^43^,^44^,^45^,^46^, in spite of the fact that for specific types of systems, e.g., gene regulatory networks, controllability can be defined in terms of the coexisting attractors (final destinations) of the system^45^. Unlike linear networked systems, controllability of a nonlinear network depends on both the network structure and the system dynamics. We speculate that the physical controllability of a nonlinear dynamical network, if it can indeed be defined, would depend on both the structural controllability and the system dynamics. At the present we still know very little about controlling complex networks hosting nonlinear dynamics, and further effort is needed to address this challenging but greatly important problem shared by a wide range of fields.

## Methods

### Control energy of one-dimensional chain model

To gain insights into how a network’s structure affects the control energy, we rewrite Eq. (3) as 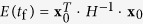, where 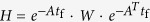. Since *H* is positive definite and symmetric like *W*, its inverse *H*^−1^ can be decomposed in terms of its eigenvectors as *H*^−1^ = *Q* · Λ · *Q*^*T*^, where *Q* = [*q*_1_, *q*_2_, …, *q*_*N*_] is composed of the orthonormal eigenvectors that satisfy *Q* · *Q*^*T*^ = *Q*^*T*^ · *Q* = *I*, and Λ = *diag*{*λ*_1_,*λ*_2_, …, *λ*_*N*_} is the diagonal eigenvalue matrix of *H*^−1^ in a descending order. Numerically, we find that *λ*_1_ is typically much larger than other eigenvalues. We thus have 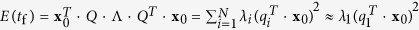.

In an undirected network, the adjacency matrix *A* is positive definite and symmetric. We can decompose *A* into the form *A* = *V* · *S* · *V*^*T*^, where the columns of *V* constitute the orthonormal eigenvectors of *A* and *S* = *diag*{*s*_1_, *s*_2_, …, *s*_*N*_} is the diagonal eigenvalue matrix of *A* in a descending order. We thus have


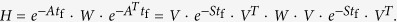


Let


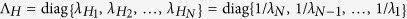


be the eigenvalue matrix of *H* in a descending order. The energy can be expressed as


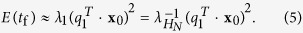


We consider a bidiretional 1D chain network and provide an analytical calculation of the relationship between control energy and chain length *L*. In the undirected chain, the adjacency matrix is defined as


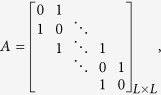


the control matrix is *B* = [1, 0, …, 0]^*T*^, and the eigenvalues and eigenvectors of *A* are, respectively,


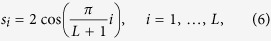



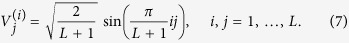


Recall that 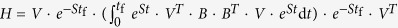. Substituting this in Eqs (6) and (7), after some algebraic manipulation, we obtain


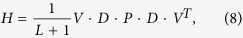


where *D* = diag{sin(*θ*), sin(2*θ*), …, sin(*Lθ*)}. and 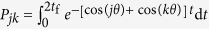 with *θ* = *π*/(*L* + 1), *j, k* = 1, …, *L*.

The Rayleigh-Ritz theorem can be used to bound *P* as:


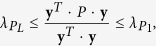


and **y** = [*y*_1_, *y*_2_, …, *y*_*L*_]^*T*^ is an arbitrary nonzero column vector, 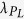 and 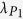 are the maximal and minimal eigenvalues of *P*, respectively. Letting *T* = 2*t*_f_, we have


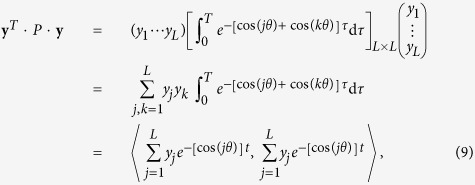


with 
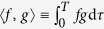
. Letting 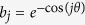 and performing a Taylor expansion on 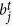 around *t* = 0, we obtain 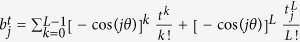, with *t*_*j*_ ∈ [0, *T*]. Now letting 
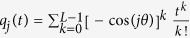
, we have 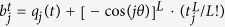. Consequently, the numerator in the Rayleigh quotient can be expressed as


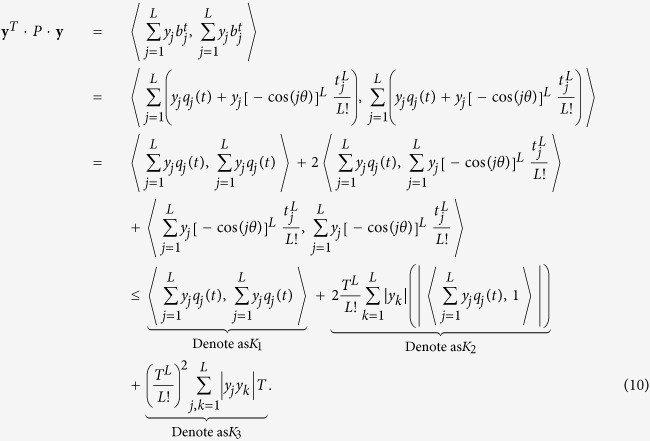


**Figure 5:**
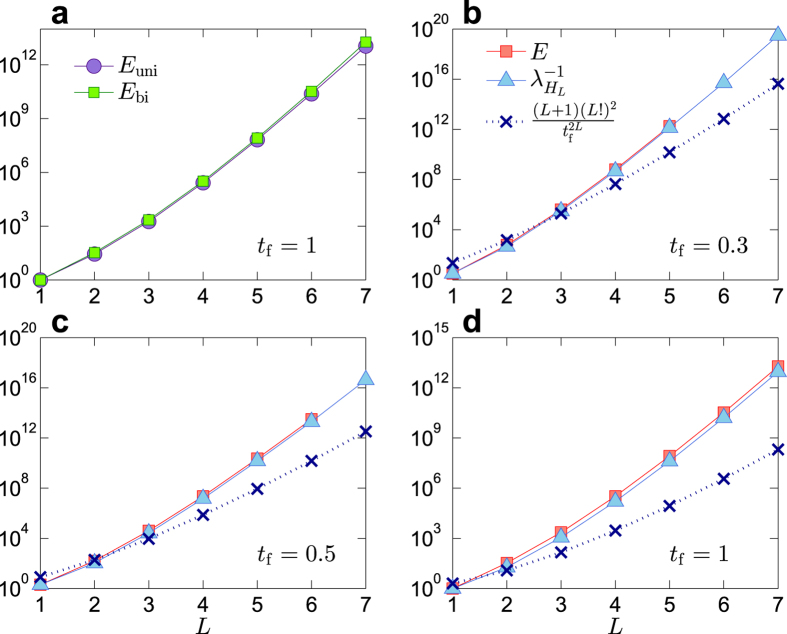
Control energy for 1D chain. (**a**) Energies required to control a unidirectional chain *E*_uni_ (purple circles) and a bidirectional one *E*_bi_ (green squares) versus chain length *L*. (**b**), (**c**) and (**d**) Control energies of bidirectional chain calculated by simulation (red squares), 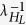 (azure triangles), and chain length *L* as shown in Eq. (12) (navy crosses) for different values of the control time *t*_f_ = 0.3, 0.5, 1, respectively.

Since **y** = [*y*_1_, *y*_2_, …, *y*_*L*_]^*T*^ is an arbitrary nonzero column vector, for each *L* and *T*, we can choose **y** = **y**_*m*_ insofar as *K*_1_ and *K*_2_ are relatively small compared with *K*_3_. We can normalize 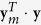 to arrive at


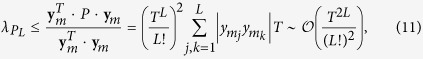


where 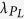 is the smallest eigenvalue of *P*. Since *P* is symmetric and positive definite, using Cholesky decomposition we can obtain its factorization^40^ as *P* = *U*^*T*^ · *U*, where *U* is the upper triangular matrix with its diagonal being the square roots of eigenvalues of *P*. Equation (8) can then be written as 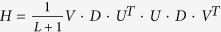. Since orthonormal transform does not change the eigenvalues of a matrix, *H* has the same eigenvalues as 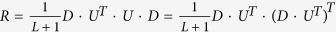. Suppose 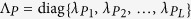 is the diagonal eigenvalue matrix of *P* in a descending order. We then have


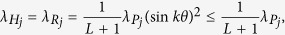


where *j* and *k* run from 1 to *L*. For arbitrary but fixed **x**_**0**_, the control energy *E(t*_f_) can be approximated as


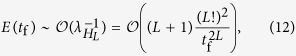


where we see that *E(t*_f_) increases faster than exponential with *L*. As shown in Fig. 5(a), the energy required to control a unidirectional 1D chain nearly overlaps with that of a bidirectional one with identical weights. From Fig. 5(b–d) we see that Eq. (12) provides a reasonably accurate estimate of the control energy.

**Figure 6:**
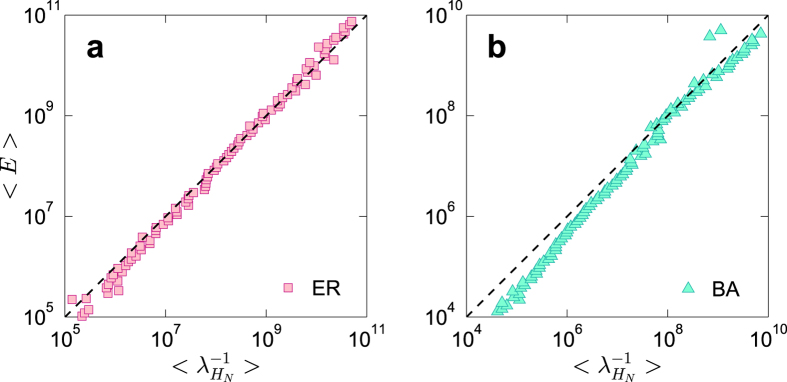
Correlation between network control energy and the smallest eigenvalue of *H*-matrix. Network size is *N* = 100, directional link probability between any pair of nodes is *P*_b_ = 0.1, and average degree is (**a**) 〈*k*〉 = 6 for ER random networks and (**b**) 〈*k*〉 = 8 for BA sale-free networks.

Furthermore, we find numerically that Eq. (5) holds for random and scale-free networks. As shown in Fig. 6, there is a strong correlation between the average network control energy, 〈*E*〉, and the smallest eigenvalue of the *H*-matrix, 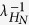, for ER random and BA scale-free networks, indicating that the network control energy is essentially determined by the smallest eigenvalue of its *H*-matrix.

### Network representation of a circuit system

**Figure 7:**
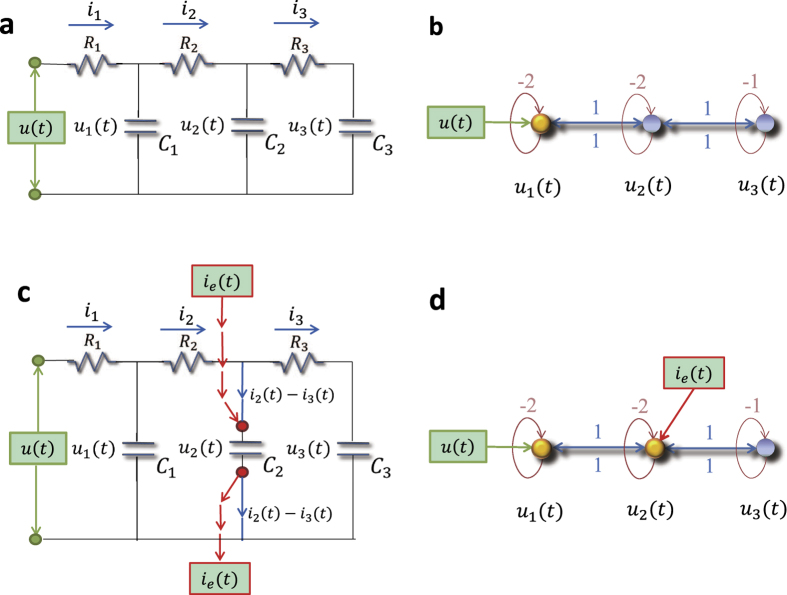
Controlling and optimizing a cascade parallel RC circuit system and the corresponding network presentation. (**a**) A cascade parallel R-C circuit with 3 resistors (*R*_1_, *R*_2_, and *R*_3_, each of resistance 1Ω) and 3 capacitors (*C*_1_, *C*_2_, and *C*_3_, each of capacitance 1F), where *u(t*) is the external input voltage, *u*_1_(*t*), *u*_2_(*t*), and *u*_3_(*t*) are the voltages on the capacitors *C*_1_, *C*_2_, and *C*_3_, and *i*_1_(*t*), *i*_2_(*t*), and *i*_3_(*t*) are the currents through the resistors *R*_1_, *R*_2_, and *R*_3_, respectively. (**b**) Network representation of the circuit in (**a**). (**c**) Circuit with an extra external current input *i*_e_(*t*) into the capacitor *C*_2_. (**d**) The extra external current input *i*_e_(*t*) serves as a redundant control input injected into node 2 of the network in (**b**). There are two driver nodes (yellow) in the network: 1 and 2.

We consider a cascade parallel R-C circuit consisting of three identical resistors and capacitors as an example to illustrate how the circuit can be abstracted into a directed network, as shown in Fig. 7. For convenience, we set *R*_1_ = *R*_2_ = *R*_3_ = *R* and *C*_1_ = *C*_2_ = *C*_3_ = *C*, and denote the currents through *R*_1_, *R*_2_, and *R*_3_ as *i*_1_(*t*), *i*_2_(*t*), and *i*_3_(*t*), respectively. The equations of the circuit are


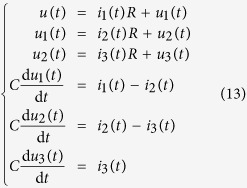


After some algebraic manipulation, we have


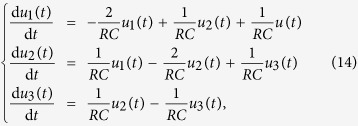


which can be written as


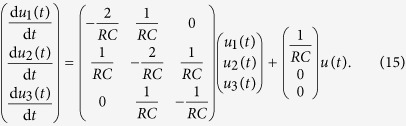


Setting *R* = 1Ω and *C* = 1*F*, we have


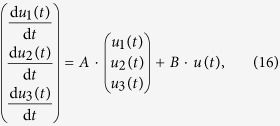


where


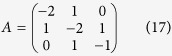


is the adjacency matrix of the network representing the circuit, and


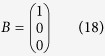


is the control input matrix. The circuit has thus been transferred into a 3-node bidirectional 1D chain network with adjacency matrix *A*.

### Implementation of extra control input in the circuit system

Without loss of generality, we inject an extra external current input *i*_e_(*t*) into the capacitor *C*_2_, and the circuit equations become:


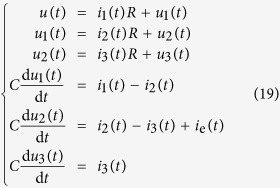


The state equations are


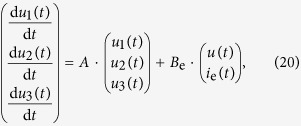


where


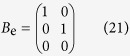


is the control input matrix of the circuit under the original control input *u(t*) on node 1 and a redundant control input *i*_e_(*t*) to node 2. Similarly, the redundant control input can be injected into any capacitor.

It is necessary to keep all other nodes unaffected while introducing exactly one extra control input into the circuit. However, any additional voltage change in any part of the circuit can lead to voltage changes on all the capacitors. A change in the current through a capacitor will not affect the currents in other components of the network, since only the time derivative of its voltage is affected. Thus, a meaningful way to introduce an extra control signal input to one node of a circuit’s network is to inject current into one particular capacitor in the circuit.

## Additional Information

**How to cite this article**: Wang, L.-Z. *et al*. Physical controllability of complex networks. *Sci. Rep.* **7**, 40198; doi: 10.1038/srep40198 (2017).

**Publisher's note:** Springer Nature remains neutral with regard to jurisdictional claims in published maps and institutional affiliations.
